# Enhancing Wildlife Trade Monitoring in the European Union—No Need to Reinvent the Wheel

**DOI:** 10.1002/ece3.72090

**Published:** 2025-08-28

**Authors:** Monica V. Biondo, Ricardo Calado

**Affiliations:** ^1^ Fondation Franz Weber Bern Switzerland; ^2^ ECOMARE—Laboratory for Innovation and Sustainability of Marine Biological Resources, CESAM—Centre for Environmental and Marine Studies, Department of Biology Santiago University Campus, University of Aveiro Aveiro Portugal

**Keywords:** European Union, monitoring, TRACES, wildlife trade

## Abstract

Biodiversity loss is mainly driven by changes in land use and overexploitation, with the commercial trade of wildlife being a smaller but still important contributor to resource depletion and species decline. The trade—including live animals, plants, fungi, and derived products—is a major economic sector valued at US$145–220 billion annually. The European Union (EU) is a key market, importing wildlife products worth approximately €100 billion. While legal trade has surged, illegal wildlife trade remains a significant transnational crime, estimated at US$20 billion annually and threatening endangered species. Monitoring wildlife trade is challenged by species‐level data gaps and inadequate regulation of many traded species, hindering conservation efforts and increasing biosecurity risks, including the transmission of zoonotic diseases. Global databases, like the United Nations Comtrade, provide insufficient species‐specific details, limiting regulatory effectiveness. The EU's database Trade Control and Expert System (TRACES) offers a powerful, underutilized tool for wildlife trade monitoring. Originally designed for biosecurity, TRACES enables real‐time tracking of wildlife imports across 90+ countries in 39 languages. It facilitates detailed data collection, cross‐border information sharing, accurate species identification, automated processing, and enhanced risk assessment. However, studies indicate that significant gaps in species identification persist, which could be mitigated through stricter enforcement and data verification. A more effective use of TRACES is needed to reinforce the EU's leadership in sustainable wildlife trade regulation, protecting endangered species while promoting ecological integrity.

## Graphical abstract

The EU plays a major role in the global wildlife trade, a sector worth over €100 billion, yet faces serious challenges due to inadequate species‐level data and regulatory gaps. Strengthening the enforcement and use of the EU's existing TRACES system could significantly improve wildlife trade monitoring, combat illegal trade, and support biodiversity conservation. Rather than developing new tools, the EU should focus on maximizing TRACES' potential for better species identification, risk assessment, and regulatory impact.

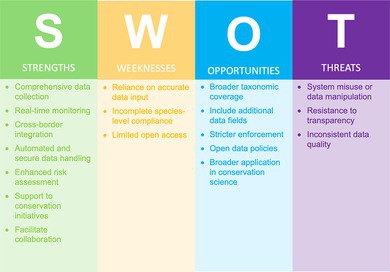

## 1 Introduction

The global wildlife trade—both legal and illegal—is a multibillion‐dollar industry that plays a major role in biodiversity loss and the unsustainable use of natural resources (Bush et al. 2014; Maxwell et al. 2016; Díaz et al. 2019; Morton et al. 2021; Fromentin et al. 2022; IPBES 2022; Hughes, Auliya, et al. 2023). It involves a wide array of live animals, plants, fungi, and derived products used for food, medicine, pets (including exotic species), and ornamentation, but it threatens the survival of many species (Symes et al. 2018; Scheffers et al. 2019; Hinsley et al. 2023). It is estimated that nearly one million animal and plant species may currently face some type of extinction risk due to a combination of factors, including habitat loss, overexploitation, climate change, and pollution (Díaz et al. 2019), and beyond conservation concerns, the trade also contributes to biological invasions, zoonotic disease outbreaks (e.g., COVID‐19, Ebola, Avian Flu), and animal welfare violations (Bezerra‐Santos et al. 2021; Rush et al. 2021; Nawtaisong et al. 2022; Li et al. 2023).

Effective monitoring tools—such as electronic permitting systems, traceability technologies, and trade databases—are essential to ensure the sustainability, transparency, and legality of wildlife trade (CITES 2016). The Convention on International Trade of Endangered Species of Wild Fauna and Flora (CITES) adopts a precautionary approach by allowing species to be listed even amid uncertainty, but inadequate trade, biological, or ecological data can leave potentially at‐risk species unlisted and legally traded without oversight (Hughes, Morton, et al. 2023). Accurate monitoring requires species‐level taxonomic identification, quantification, traceability of origin and supply chains, and clear distinction between wild, captive‐bred, or artificially propagated sources—criteria rarely met in practice. Legal trade is often assumed to be sustainable (Hughes, Morton, et al. 2023), yet many species remain unregulated due to data gaps that hinder risk assessments and policy responses (Fukushima et al. 2020; Stephenson et al. 2022; Hughes, Morton, et al. 2023; Marshall et al. 2025).

Existing trade databases reflect these limitations. The UN Comtrade database provides only aggregated data using broad Harmonized System (HS) codes based on value and weight, lacking species‐specific details and origin information (Drinkwater et al. 2021; United Nations 2025), both of which are crucial for conservation planning (Cawthorn and Mariani 2017). In contrast, the US Law Enforcement Management Information System (LEMIS) offers more detailed records to support enforcement and conservation, but often aggregates data at broad taxonomic levels, limiting species‐level monitoring (Marshall et al. 2025). In light of these shortcomings, a critical review of current data sources—particularly the potential of tools like TRACES is—urgently needed to evaluate their effectiveness and inform better governance of wildlife trade (Table S1).

### 1.1 Legal Trade

The global wildlife trade is a major economic sector, valued at an estimated US$145–220 billion annually, driven largely by commodities such as seafood, furniture, and fashion products (Andersson et al. 2021). The European Union (EU), one of the largest global economies (EUROSTAT 2024), is a key market and transit region, importing around €100 billion in wildlife products, mainly fishery goods, timber, and ornamental species (CITES 2022; UNDOC 2024; EC 2025; TRAFFIC 2025a). International legal wildlife trade has surged, increasing 500% since 2005 and 2000% since the 1980s, partly due to more species being captive‐bred or ranched (IPBES 2020). These estimates exclude timber and commercial fisheries, indicating the actual trade volume is considerably larger (Díaz et al. 2019).

### 1.2 Illegal Trade

The Convention on International Trade in Endangered Species of Wild Fauna and Flora (CITES) provides a legally binding framework for regulating trade in over 40,000 species across 185 countries. International trade without the necessary permits is illegal. CITES oversees an estimated US$1.8 billion in legal animal and US$9.3 billion in plant trade annually, though this reflects only a fraction of the global wildlife trade (CITES 2022). Illegal trade in CITES‐listed species persists, undermining conservation efforts, with the EU as a major market and destination. In 2023, the EU recorded the highest number of seizure incidents and one of the largest volumes of specimens seized to date. The main countries of origin for CITES‐listed exports seized in the EU were Thailand, the USA, Ukraine, mainland China, and India (TRAFFIC 2025b). CITES faces limitations due to gaps in trade, biological, and ecological data, which hinder effective listing and monitoring (Stephenson et al. 2022). Many species receive limited policy attention, reducing conservation efficacy (Hinsley et al. 2024).

The EU consistently ranks among the top global regions for wildlife trade volume and value (CITES 2022). The EU Wildlife Trade Information Exchange (EU‐TWIX) supports monitoring and enforcement against illegal CITES trade within the EU and has been proposed for broader monitoring. However, it lacks data on non‐CITES‐listed species (Cardoso et al. 2024) and is hampered by inconsistent implementation and data quality across member states (EC 2022).

Domestic legislation is vital in combating illegal trade. The US Lacey Act, for example, bans the import of wildlife or plants harvested illegally under the laws of their origin country, even if international trade is otherwise permitted (U.S. Government 1900, as amended). Such laws complement international frameworks like CITES by enhancing enforcement. Similar criminalization measures are under discussion in the EU, as they would not require extraterritorial jurisdiction and would align with existing EU policies and legislation (EC 2025).

Illegal wildlife trade significantly contributes to population declines in endangered species such as elephants and rhinos (Symes et al. 2018; Hinsley et al. 2024; UNDOC 2024). Generating an estimated US$7–23 billion annually (figures based on seizure data and likely underestimated) this trade ranks among the largest transnational criminal markets (UNEP and INTERPOL 2016; INTERPOL 2023), though it remains a fraction of the legal wildlife trade.

## 2 TRACES: An Existing Tool for Enhanced Wildlife Trade Monitoring in the EU

The Trade Control and Expert System (TRACES), established by the EU in 2004, is a digital certification platform originally designed for biosecurity purposes. It records detailed information on consignments of live animals, animal products (e.g., meat, dairy, eggs, hides, leather goods), plants, food, feed, and derived products (e.g., wool, reptile skins, ornamental wood), including origin, species, quantity, transport, and sanitary or phytosanitary status to support traceability and border control. Although its primary goal is biosecurity, many of its existing features already support the monitoring of wildlife trade.

TRACES has a global outreach, integrating data from over 90 countries and operating in at least 39 languages. However, it is limited to trade into and within the EU member states and does not capture transactions between non‐EU countries. This limitation highlights the need for complementary systems to monitor global wildlife trade in a more comprehensive way.

Overall, TRACES holds a largely untapped potential as a species‐level monitoring and enforcement tool. In 2023 alone, the system handled over 2.7 million customs health declarations of the 4.7 million official documents issued (EC 2024).

Despite its extensive reach and data volume, TRACES remains largely underutilized in scientific research supporting decision‐making, partly due to difficult accessibility. While certain taxa, such as marine ornamental fishes, are relatively well represented in the TRACES database (Biondo et al. 2024), others—as for example amphibians—are often aggregated under broad categories such as ‘other live animals’. It is important to highlight that TRACES covers all animals, including both vertebrates and invertebrates, but the taxonomic resolution varies across groups. Identifying which amphibian species are traded typically requires submitting a formal data request, including a statement of purpose, followed by a time‐consuming data analysis process to extract species‐level information from these aggregated codes. Overall, data granularity may not always exist on TRACES database for some taxonomic groups. Only a few studies to date have highlighted its potential to monitor the wildlife trade. For instance, a quantitative import risk analysis (QIRA) model incorporating TRACES data was used to assess the risk of Lumpy Skin Disease entering France, illustrating the system's adaptability for managing disease risks (Saegerman et al. 2019). Other studies on cattle transport within Europe underscored its operational scale (Padalino et al. 2021; Bachelard 2022). Yet, a more recent analysis of marine ornamental fish imports found that 30% of shipments lacked species‐level identification, exposing major data gaps in wildlife trade monitoring (Biondo and Calado 2021; Biondo et al. 2024). The shortcomings associated with TRACES point to broader challenges in data quality and enforcement. By enhancing TRACES through standardized species‐level reporting, improved verification protocols, and better integration with conservation‐focused monitoring, the platform could significantly strengthen wildlife trade oversight, not only at EU level but globally.

## 3 Challenges and Opportunities of TRACES for Wildlife Trade Regulation: Addressing the Pros and Cons

**FIGURE 1:**
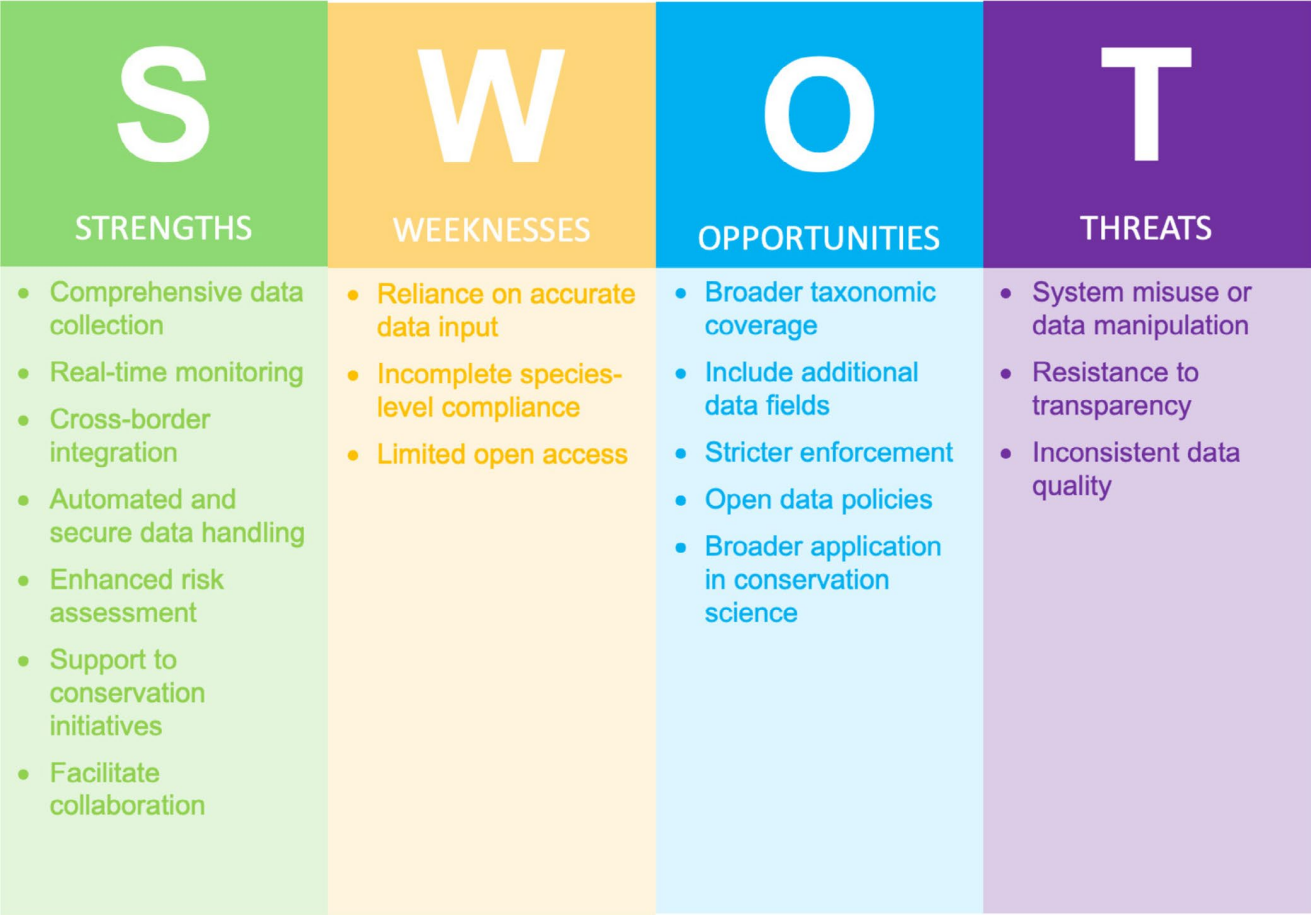
SWOT analysis of the European Trade Control and Expert System (TRACES).

The SWOT analysis presented identifies both the strengths and limitations of the current TRACES platform in the context of wildlife trade monitoring and sustainability assurance (Figure 1).

### 3.1 Strengths

- Comprehensive data collection: TRACES records detailed data on species, quantities, and invasiveness (rapid spread and disruption of habitats outside the species native range), aiding in risk assessments and improving regulatory oversight.
- Real‐time monitoring: Enables the detection of suspicious activity promptly, facilitating timely enforcement of trade regulations.
- Cross‐border integration: Harmonized across all EU member states and associated countries, TRACES standardizes reporting consistency and reduces the risks of fraudulent and illicit trade.
- Automated and secure data handling: Digital format reduces human error, enhances transparency, and strengthens detection of fraudulent practices. The platform's current functionality already supports bulk data uploads via CSV and Excel formats (TRACES 2025).
- Enhanced risk assessment: Used in analyzing trade patterns and species trends, for example, marine ornamental fishes (Biondo and Burki 2019; Biondo et al. 2024), with potential for expansion to other taxa to facilitate better identification of high‐risk species and targeted conservation efforts.
- Support to conservation initiatives: Accurate trade documentation via TRACES supports the EU's broader conservation goals, including the Biodiversity Strategy 2030 (EC 2020) and the Action Plan against Wildlife Trafficking (EC 2022). The EU's robust legal framework combines CITES implementation, strict wildlife trade regulations, and enforcement tools such as EU‐TWIX, while fostering cooperation with global bodies like the International Criminal Police Organization (INTERPOL), the World Customs Organization (WCO), and the United Nations Office on Drugs and Crime (UNDOC).
- Facilitate collaboration: Connects data between customs, regulatory authorities, and logistic firms, supporting coordinated global action.

### 3.2 Weaknesses

- Reliance on accurate data input: Effectiveness depends heavily on accurate and honest reporting by actors in the trade chain (e.g., customs brokers, wholesalers). Errors or omissions—accidental or intentional—can undermine data quality.
- Incomplete species‐level compliance: While species‐level reporting is supported, compliance with this requirement is inconsistent and needs stronger enforcement to ensure accurate data collection and safeguard data granularity (Regulation (EU) 2016/429 2016).
- Limited open access: TRACES is not openly accessible to the public or researchers, but data can be provided upon request. If open access is unlikely to be implemented, clear guidelines on data request procedures and EU/non‐EU access conditions are needed.

### 3.3 Opportunities

- Broader taxonomic coverage: Expand monitoring to include all wildlife taxa (e.g., vertebrates, invertebrates, plants, and fungi) to ensure comprehensive trade assessments and ecological risk evaluations.
- Include additional data fields: Among others, place of origin (source) and details of the supply chain. Expanding TRACES would impose minimal burden on exporters, given the system's established familiarity and existing user infrastructure.
- Stricter enforcement: Implementing stronger legal sanctions for misreporting or misconduct could improve compliance and accountability.
- Open data policies: Ensure that all TRACES operators cooperate, share knowledge openly, and contribute to making data truly FAIR (Findable, Accessible, Interoperable, and Reusable) improving data accessibility for global conservation (Wilkinson et al. 2016).
- Broader application in conservation science: Applying TRACES data across more research areas could support the identification of high‐risk species and inform conservation priorities more effectively.

### 3.4 Threats

- System misuse or data manipulation: If regulatory controls are weak or unevenly applied, actors may attempt to exploit the system, undermining its integrity.
- Resistance to transparency: Limited public access and possible reluctance to expand transparency may hinder collaborative oversight and civil society engagement.
- Inconsistent data quality: Disparities in reporting practices and lack of standardization outside the EU may reduce the reliability of integrated datasets.

## 4 Conclusion

Existing tools for monitoring wildlife trade fall short in data precision, taxonomic granularity, and system‐wide consistency. Without improvements in data quality and interoperability—especially for non‐CITES‐listed species—monitoring and regulation will remain insufficient.

Legal wildlife trade is often perceived as sustainable, yet legality does not guarantee sustainability, neither by definition nor in practice. This misconception is widespread and problematic, mostly because many legally traded species are still poorly monitored or biologically vulnerable to collection. Moreover, the sheer volume of legal imports further challenges the assumption that trade is sustainable. Illegal trade is widely recognized as unsustainable and often overlaps with legal trade, exploiting loopholes and deficiencies in existing monitoring systems. From a conservation standpoint, any form of unsustainable trade—regardless of its legal status—requires closer scrutiny and regulatory attention. TRACES offers a strategic opportunity for the EU to enhance cross‐border regulatory processes. Its established infrastructure across EU member states and associated countries, along with widespread user familiarity, positions it well for integration with existing legal frameworks at relatively low cost. However, improving access to relevant data remains essential. Enhancing TRACES to strengthen species‐level reporting would support more robust baseline data and improve oversight of legal wildlife trade. Greater transparency in the legal wildlife trade will enable faster detection and exposure of activities linked to illegal wildlife trade.

With strategic investment and strong administrative commitment, the EU can reinforce its position as a global leader in sustainable wildlife trade regulation. TRACES can underpin this role if its future development prioritizes stricter compliance, enhanced transparency, and policy reforms that address current gaps, such as ensuring strict taxonomic accuracy to secure data granularity.

To realize this potential, the EU should strengthen TRACES enforcement, improve species‐level reporting, and promote consistent, comprehensive data collection and sharing. These measures would significantly enhance the EU's ability to monitor legal trade, detect irregularities, and support biodiversity conservation—without the need to reinvent the wheel.

## Author Contributions

**Monica V. Biondo:** conceptualization (equal), investigation (equal), methodology (equal), project administration (equal), visualization (lead), writing – original draft (lead), writing – review and editing (equal). **Ricardo Calado:** conceptualization (equal), investigation (equal), methodology (equal), project administration (equal), resources (equal), visualization (supporting), writing – original draft (supporting), writing – review and editing (equal).

## Conflicts of Interest

The corresponding author works for a conservation NGO, Fondation Franz Weber. The other author declares no conflicts of interest.

## Supporting information


**Table S1:** Non‐exhaustive list of global and regional systems for monitoring legal and illegal trade in CITES‐ and non‐CITES species.

## Data Availability

The authors have nothing to report.
