# Supplementary material for: Enhancing Wildlife Trade Monitoring in the European Union—No Need to Reinvent the Wheel
Source: Ecol Evol. 2025 Aug 28;15(9):e72090. doi: 10.1002/ece3.72090 (PMC12394888; doi:10.1002/ece3.72090)
Supplement: Supplementary file 1 — Table S1: Non‐exhaustive list of global and regional systems for monitoring legal and illegal trade in CITES‐ and non‐CITES species. [file ECE3-15-e72090-s001.docx]

**SUPPLEMENTARY MATERIAL Table S1**

**Non-exhaustive list of global and regional systems for monitoring legal and illegal trade in CITES- and non-CITES species**

| **Name of database** | **Description** | **Spatial extent** | **Taxonomic extent** | **Granularity** | **# trade records** | **Main limitations** | **Accessibility** |
| --- | --- | --- | --- | --- | --- | --- | --- |
| **LEGAL TRADE** | | | | | | | |
| **GLOBAL** | | | | | | | |
| **UN Comtrade** United Nations’ global repository for international trade statistics | Global database of international trade statistics maintained by the United Nations since 1962. Provides trade data in kilograms, tons, units, dollars. <https://comtrade.un.org> | Covers plants, animals, fungi, and microorganisms, but not taxonomically labeled.  Harmonized System (HS), with 6-digit standard codes (can be extended nationally). | Aggregated by country, year, product, value and weight. Uses commodity codes, not scientific names. | Harmonized System (HS) codes | ~3,000 million records (as of 2025), from nearly 200 countries.^[[1]](#footnote-1)^ | No taxonomic or ecological data; cannot distinguish between wild, cultivated, or synthetic sources. | Registration required |
| **CITES** Convention on International Trade of Endangered Species | Tracks imports/exports of wildlife and wildlife products listed on its appendices^[[2]](#footnote-2)^, helping enforce the CITES Convention and prevent illegal or unsustainable trade since 1975. [www.cites.org](http://www.cites.org); [https://trade.cites.org](https://trade.cites.org/) | Only species which are listed in the Annexes of the Convention. | All species excluding fungi and microorganisms. | Species-level | ~23 million records (as of October 2023).^[[3]](#footnote-3)^ Adding 1 million records a year. | Trade data is self-reported by each country without automatic cross-checks, often resulting in timing discrepancies and gaps due to late or missing reports. | Open access |
| **REGIONAL** | | | | | | | |
| **U.S. LEMIS** Law Enforcement Management Information System | Official U.S. Fish and Wildlife Service (USFWS) database tracking wildlife and wildlife products imported into the U.S. since 1983.  [www.fws.gov](http://www.fws.gov) | Both **CITES-listed** and **non-CITES** species imported into the U.S. | All species excluding fungi and microorganisms. | Varies by record: species, genus, family, or species group. | >2 million shipments, representing more than 60 biological classes and 3.2 billion live organisms (2000-2014).^[[4]](#footnote-4)^ | U.S.-only data with no visibility into non-U.S. trade; post-2014 data is not public and may be inconsistent or require cleaning. | Data for 2000-2014 publicly available;^[[5]](#footnote-5)^ newer data via Freedom of Information Act. |
| **TRACES** Trade Control and Export System | Operated by the Directorate-General for Health and Food Safety (DG SANTE) of the European Commission, this official digital platform certifies sanitary and phytosanitary requirements for import, export, and intra-EU trade; since 2004, it facilitates traceability, risk management, and streamlines control of goods subject to veterinary and phytosanitary regulations across EU and non-EU countries.  <https://food.ec.europa.eu/horizontal-topics/traces_en> | Live animals, animal products, food, feed, and plants imported into the EU. | All species including in parts fungi. | Often **commodity-based; s**pecies-level data may be available upon request and detailed analysis**.** | 2.7 million Common Health Entry Documents (CHED) of the 4.7 million official documents issued in 2023.^[[6]](#footnote-6)^ | May lack species-level identification and 'wild/captive' status; some records may contain species details but require analysis to extract them. | Upon request |
| **ILLEGAL TRADE** | | | | | | | |
| **GLOBAL** | | | | | | | |
| **WiTIS**  TRAFFIC Wildlife Trade Portal | Database of illegal wildlife trade incidents (including seizures) collated from publicly accessible sources; established in 2012.  [www.wildlifetradeportal.org](http://www.wildlifetradeportal.org) | Global data from over 120 countries. | Hardly any reporting on invertebrate or fungi seizures. | Records individual incidents with details on species, commodity type, quantities, locations, dates, and enforcement outcomes. | ~720,000 records; regularly updated.^[[7]](#footnote-7)^ | Limitations including inconsistent country reporting causing underrepresentation, variable data accuracy due to differing standards, some features requiring user registration, and irregular update frequency affecting data timeliness. | Registration required |
| **REGIONAL** | | | | | | | |
| **EU TWIX** Wildlife Information eXchange | Tracks illegal trade in species listed under CITES and the EU Wildlife Trade Regulations; active since 2005.  [www.eu-twix.org](http://www.eu-twix.org) | Data from all 27 EU member states, and various additional European countries responsible for implementing CITES through their national legislation. | Species covered by the EU Wildlife Trade Regulations^[[8]](#footnote-8)^ and CITES, which predominantly include vertebrates and certain plant species. | Species-level where possible, with details on species, genus, or family involved in seizures and trade incidents. | 91,000 records.^[[9]](#footnote-9)^ | Invertebrates and fungi are less frequently reported in seizure data. | Access available to European wildlife law enforcement officials upon registration. |
| **WCN**  Wildlife Confiscations Network | A U.S. network linking law enforcement with accredited care facilities to house and rehabilitate confiscated live wildlife since 2023. Case information originates from U.S. Fish and Wildlife Services (USFWS) seizure records stored in LEMIS.  [www.aza.org/wta-confiscations-network](http://www.aza.org/wta-confiscations-network) | Initially Southern California, now expanding toward national coverage. | All live taxa seized in wildlife trafficking cases. | Case-level records with species identification, quantity, and disposition. | Of the 834 live wildlife seizures by USFWS in 2015–2019, WCN has handled ~135 cases and placed over 4,100 animals as of 2025.^[[10]](#footnote-10)^ | Focuses only on live animals needing care; not a comprehensive seizure database; data not standardized for research. | Not publicly searchable; summaries shared in reports and press releases. |

1. <https://comtrade.un.org/labs/data-explorer/About.html> [↑](#footnote-ref-1)
2. <https://cites.org/eng/app/index.php> [↑](#footnote-ref-2)
3. [https://trade.cites.org/cites_trade_guidelines/en-CITES_Trade_Database_Guide.pdf](https://trade.cites.org/cites_trade_guidelines/en-CITES_Trade_Database_Guide.pdf?utm_source=chatgpt.com) [↑](#footnote-ref-3)
4. <https://www.nature.com/articles/s41597-020-0354-5> [↑](#footnote-ref-4)
5. <https://zenodo.org/records/3565869> [↑](#footnote-ref-5)
6. <https://food.ec.europa.eu/horizontal-topics/traces/information-material/traces-annual-report-2023_en> [↑](#footnote-ref-6)
7. <https://www.traffic.org/what-we-do/thematic-issues/trade-monitoring/> [↑](#footnote-ref-7)
8. <https://eur-lex.europa.eu/legal-content/EN/TXT/?uri=uriserv%3AOJ.L_.2023.133.01.0001.01.ENG&toc=OJ%3AL%3A2023%3A133%3ATOC> [↑](#footnote-ref-8)
9. <https://www.traffic.org/site/assets/files/3792/eu-twix-leaflet-2024-en.pdf> [↑](#footnote-ref-9)
10. <https://assets.speakcdn.com/assets/2332/wta_fws-seizures-data_final.pdf> [↑](#footnote-ref-10)
